# Nucleotide and phylogenetic analyses of the *Chlamydia trachomatis ompA *gene indicates it is a hotspot for mutation

**DOI:** 10.1186/1756-0500-5-53

**Published:** 2012-01-20

**Authors:** Brian W Brunelle, George F Sensabaugh

**Affiliations:** 1 Food Safety and Enteric Pathogens Research Unit, USDA, ARS, National Animal Disease Center, Ames, IA 50010, USA; 2 Division of Infectious Diseases, School of Public Health, University of California, Berkeley, CA 94720, USA

**Keywords:** *Chlamydia trachomatis*, Evolution, MOMP, *ompA*, Phylogenetics

## Abstract

**Background:**

Serovars of the human pathogen *Chlamydia trachomatis *occupy one of three specific tissue niches. Genomic analyses indicate that the serovars have a phylogeny congruent with their pathobiology and have an average substitution rate of less than one nucleotide per kilobase. In contrast, the gene that determines serovar specificity, *ompA*, has a phylogenetic association that is not congruent with tissue tropism and has a degree of nucleotide variability much higher than other genomic loci. The *ompA *gene encodes the major surface-exposed antigenic determinant, and the observed nucleotide diversity at the *ompA *locus is thought to be due to recombination and host immune selection pressure. The possible contribution of a localized increase in mutation rate, however, has not been investigated.

**Results:**

Nucleotide diversity and phylogenetic relationships of the five constant and four variable domains of the *ompA *gene, as well as several loci surrounding *ompA*, were examined for each serovar. The loci flanking the *ompA *gene demonstrated that nucleotide diversity increased monotonically as *ompA *is approached and that their gene trees are not congruent with either *ompA *or tissue tropism. The variable domains of the *ompA *gene had a very high level of non-synonymous change, which is expected as these regions encode the surface-exposed epitopes and are under positive selection. However, the synonymous changes are clustered in the variable regions compared to the constant domains; if hitchhiking were to account for the increase in synonymous changes, these substitutions should be more evenly distributed across the gene. Recombination also cannot entirely account for this increase as the phylogenetic relationships of the constant and variable domains are congruent with each other.

**Conclusions:**

The high number of synonymous substitutions observed within the variable domains of *ompA *appears to be due to an increased mutation rate within this region of the genome, whereas the increase in nucleotide substitution rate and the lack of phylogenetic congruence in the regions flanking *ompA *are characteristic motifs of gene conversion. Together, the increased mutation rate in the *ompA *gene, in conjunction with gene conversion and positive selection, results in a high degree of variability that promotes host immune evasion.

## Background

*Chlamydia trachomatis *is an obligate intracellular bacterium that is the most frequently reported bacterial sexually transmitted disease in the United States [1], as well as the leading cause of infectious blindness worldwide [2]. Strains of *C. trachomatis *are differentiated into serovars based on the serospecificity of the major outer membrane protein (MOMP) that constitutes over 60% of all the surface-exposed proteins [3] and functions as a porin [4]. Serovars A-C primarily infect ocular tissue, serovars D-K typically infect urogenital tissue, and serovars L1-L3 infect lymphatic tissue. There appears to be a very high level of nucleotide identity across the genomes of the serovars [5,6] as even the most divergent genomes are over 99% identical in sequence [7]. Additionally, genomic data has demonstrated that the phylogenetic pattern of the serovars correlates with tissue tropism [8-10].

**Figure 1:**
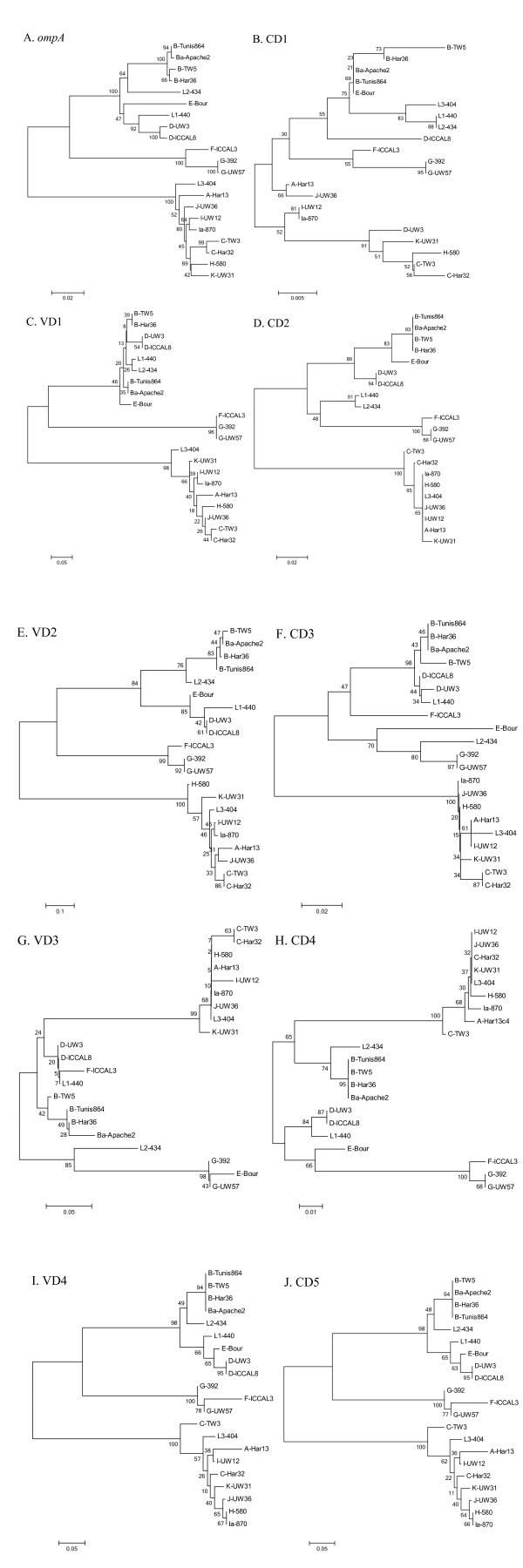
**Phylogenetic analyses of the *C. trachomatis ompA *gene and individual constant and variable domains**. Phylogenetic trees of the *C. trachomatis ompA *gene (A), as well as the five constant (CD1-5) and four variable (VD1-4) domains of the *ompA *gene (B-J), were constructed using the Maximum Composite Likelihood neighbor-joining method with pairwise deletions of alignment gaps. Results of 1000 bootstrap replicates are reported for each node.

In contrast to the low level of nucleotide variation observed in most of the genome, the *ompA *gene that encodes MOMP is characterized by a very high level of variability as nearly 30% of the coding sequence exhibits polymorphisms [11]. For comparison, the *porB *gene in *C. trachomatis *also encodes a surface-exposed porin that elicits neutralizing antibodies [12,13], but it only varies at 1% of its nucleotide sites [8]. MOMP has four variable domains that encode the surface-exposed epitopes and contain the amino acid substitutions that define serospecificity; these are separated by five membrane-spanning constant domains that are considerably more conserved than the variable domains, but are nevertheless more polymorphic than the majority of the genes in the genome. Phylogenetic characterization of the *ompA *gene exhibits a pattern of strongly supported groupings that differ markedly from the tissue tropism [11,14]: serovars A, C, H, I, J, K, and L3 comprise one group; F and G a second; D, E, and L1 a third; and B and L2 a fourth (see Figure 1A).

The discordance between the *ompA *gene tree and trees from other *C. trachomatis *loci is considered to be due to intra-species recombination [8,9,15-17]. Recombination is thought to be rare in *C. trachomatis *due to the limited opportunity other bacteria, plasmids, or exogenous DNA have to enter a chlamydial inclusion [18], but genomic sequence data indicate that intra-species recombination has occurred in 8 genes in the genome, including *ompA*, and may have occurred in as many as 47 additional genes [17]. There is evidence that recombination has acted on portions of the *ompA *gene to create mosaic MOMP structures [19-22], as well as on the entire *ompA *gene in order to replace the MOMP of an isolate [8,9,16]. Further support that chlamydia has the means and mechanisms to acquire and stably incorporate exogenous DNA is the presence of a genomic island containing a tetracycline-resistance gene found in the chromosome of several swine isolates of *Chlamydia suis *[23]. In addition to recombination, diversity generated in MOMP can be attributed to the positive selection of non-synonymous substitutions that lead to beneficial amino acid replacements, especially in a gene that encodes an antigenic outer membrane protein.

It is currently unknown, however, if the increase in nucleotide diversity in *ompA *is solely due to recombination and positive selection. In order to establish if there are additional factors involved in the generation of *ompA *diversity, we examined the nucleotide and phylogenetic associations of the five constant and four variable domains of the *ompA *gene, as these regions are under different immune and functional constraints. We also evaluated the relationships of the loci surrounding the *ompA *gene. These results demonstrate that the synonymous nucleotide diversity generated in the *ompA *gene cannot be explained by recombination and positive selection alone, and indicate that this region is prone to increased mutations compared to other regions in the genome. The data also show that the increased nucleotide substitution rate and the lack of phylogenetic associations in the regions proximal to the *ompA *gene are characteristic motifs of gene conversion.

## Results

**Figure 2:**
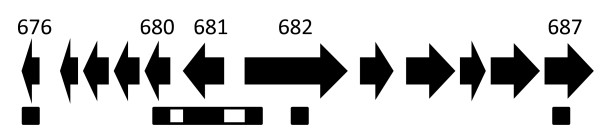
**Schematic representation of the loci under investigation**. The following eight regions were used in the study and are designated by their gene number and name: CT676 (hypothetical gene), CT680 (*rs2*), CT680-1 (intergenic region), CT681 (*ompA*), CT681-2 (intergenic region), CT682 (5' end; *pbpB*), CT682 (middle; *pbpB*), and CT687 (*yfhO2*). The arrows in the map represent the gene size and distance relative to each other; the boxes under the arrows represent the regions that were sequenced (black boxes are coding regions and open boxes are intergenic regions). Specific details on nucleotide region sizes and distances are in Table 1.

Twenty-one strains representing at least one of each serovar were used in this study (A/Har-13, B/Tunis 864, B/TW-5, B/Har-36, Ba/Apache 2, C/TW-3, C/Har-32, D/UW-3, D/IC-Cal 8, E/Bour, F/IC-Cal 3, G/UW-57, G/392, H/580, I/UW-12, Ia/870, J/UW-36, K/UW-31, L1/440, L2/434, L3/404). These are all lab-adapted strains that are common in the literature, and 18 of them were previously used to characterize regions throughout the genome [8]. These strains were sequenced at the following eight loci: CT676 (hypothetical gene), CT680 (*rs2*), CT680-1 (intergenic region), CT681 (*ompA*), CT681-2 (intergenic region), CT682 (5' end; *pbpB*), CT682 (middle; *pbpB*), and CT687 (*yfhO2*) (Figure 2).

### Sequence diversity in *ompA *and flanking regions

To assess the relationships of the *ompA *flanking regions, seven loci totaling ~2.7 kb within 10 kb of either side of the *ompA *gene were sequenced and analyzed. These included the entire contiguous non-coding regions on the 5' and 3' end of the *ompA *gene, as well as neighboring coding loci (Table 1). The rate of substitutions at both non-synonymous (d_N_) and synonymous sites (d_S_) in these genomic regions increase as they approach the *ompA *gene (Table 1). The ratio of transition to transversion events (ts/tv) in housekeeping loci outside of the *ompA *region of the genome is 10/1, but this rapidly decreases with proximity to *ompA *to a ratio of 1/1 (Table 1). Usually, the ts/tv ratio is biased towards transitions as these are biochemically simpler changes (purine to purine, and pyrimidine to pyrimidine), as was observed in the *C. trachomatis *housekeeping regions. A larger number of transversions, however, is indicative of a region more susceptible to mutation, though the *ompA *region may be biased due to saturation of nucleotide diversity [24]. Also, there are a number of insertion/deletion sites (indels) within the adjacent non-coding regions of *ompA *(10-12% of the nucleotide sites). Together, these data demonstrate that the percent of variable nucleotide sites and nucleotide diversity increase monotonically as *ompA *is approached.

**Table 1:** Nucleotide analyses of the *ompA *gene and surrounding loci

Loci	Gene	Distance from *ompA*^A^	#nt	#Δ nt sites	%Δ nt sites	d_N_	d_S_	d_N_/d_S_	ts/tv
676	CT676	4090-3803	347	16	4.61	0.003	0.065	0.05	8.6

680	*rs2*	781-370	412	40	9.71	0.015	0.112	0.13	2.9

680-1		369-1	399	54	13.53	----	----	----	2.1

681	*ompA*	----	1194	341	28.56	0.076	0.419	0.18	1.0

681-2		1-607	608	47	7.73	----	----	----	1.0

682^B^	*pbpB*	608-810	208	15	7.21	0.005	0.082	0.06	2.6

682^C^	*pbpB*	1332-1772	442	17	3.85	0.004	0.030	0.13	3.8

687	*yfhO2*	8873-9229	357	9	2.52	0.002	0.026	0.08	----^D^

HK^E^			3096	23	0.74	0.001	0.002	0.61	10.0

^A^Distance based on serovar D/UW-3 genome

^B^5' end of gene

^C^Middle of gene

^D^All 9 polymorphisms were transitions

^E^Housekeeping gene data from Brunelle and Sensabaugh, 2006 [8]

### Sequence diversity within *ompA *constant and variable domains

To compare the five constant domains within the *ompA *gene to the four interspersed variable domains [25], these nine regions were examined at the nucleotide level (Table 2). The five constant domains comprise a total of 927 nucleotide sites, 155 of which are variable (16.7%). Although the d_N_/d_S _ratio (0.04) is comparable to regions outside of *ompA*, the individual d_N _and d_S _value are each equal-to-greater than that observed in the adjacent loci. In contrast, 186 of the 267 (69.7%) total nucleotide sites in the four variable domains are variable, which is reflected in the elevated d_N_, d_S_, and d_N_/d_S _values compared to the constant domains. The considerably reduced rate of amino acid replacements in the constant regions implies they have been under a different level of selection; nonetheless, the constant domains have a high level of sequence diversity compared to regions outside of the *ompA *gene.

**Table 2:** Nucleotide analyses of the constant (CD#) and variable (VD#) domains of the *ompA *gene

	# nt	#Δ nt sites	%Δ nt sites	d_N_	d_S_	d_N_/d_S_	ts/tv
CD1	255	19	7.45	0.008	0.092	0.09	1.6

CD2	165	43	26.06	0.024	0.927	0.03	2.7

CD3	189	52	27.51	0.019	0.596	0.03	6.3

CD4	150	26	17.33	0.014	0.421	0.03	2.4

CD5	168	15	8.93	0.004	0.123	0.03	2.1

CD1-5	927	155	16.72	0.013	0.314	0.04	2.0

VD1	66	58	87.88	0.797	0.962	0.83	0.4

VD2	66	51	77.27	0.640	0.983	0.65	0.9

VD3	42	19	45.24	0.135	0.491	0.27	0.9

VD4	93	58	62.37	0.281	0.648	0.43	1.1

VD1-4	267	186	69.66	0.404	1.204	0.34	0.7

*ompA*	1194	341	28.56	0.076	0.419	0.18	1.0

### Phylogenetic analysis of *ompA *and flanking regions

Individual phylogenetic trees were constructed for *ompA *(Figure 1A) and the seven regions flanking the *ompA *gene (Additional file 1) to assess if comparable or distinct associations existed between the *ompA *gene and the neighboring loci; differing phylogenetic topologies would be indicative of recombination. The phylogenetic trees were all different between the CT676 locus, the non-coding regions immediately adjacent to either side of *ompA *(CT680-1 and CT681-2), and the two different portions of the CT682 coding region. In addition, none of these regions were congruent with the phylogeny established for either *ompA *or that based on tissue tropism [8,9,16,26]. Interestingly, serovars L1-L3 grouped together in the trees from the flanking loci, but were separated only in the *ompA *tree. The lack of overall phylogenetic congruence observed in *ompA *and its neighboring regions can be ascribed to recombination.

Indels are informative events and can serve as valuable phylogenetic markers because there is a low probability that such events would occur in an identical region of two separate strains independently. Excluding single nucleotide indels, as well as those found in a repetitive stretch of a single nucleotide base (e.g. poly-T tracts) that could be susceptible to slip-strand mispairings, the indels in the flanking non-coding regions of *ompA *were examined. The non-coding region preceding the 5' end (CT681-2) had a 21 bp deletion in strains B/Har36, B/TW5, F/IC-CAL3. Since it was not found in B/Tunis 864 or Ba/Apache2, this indicates an association among these otherwise divergent strains not seen in any of the previous phylogenetic analyses. The 2, 3, 4, 6, and 9 bp indels in the non-coding region following the 3' end of *ompA *(CT680-1) were all congruent with the phylogenetic relationship of the region. In addition, there was a tandem 8 bp motif (TATTAGAA) in A/Har13 immediately adjacent to the stop codon of *ompA*. One 8 bp sequence is found in all the strains in this location, but the insertion in strain A/Har13 was apparently due to a duplication event. This region was sequenced from a different strain of the same serovar, A/Har1, and was found to have the same octamer repeat. This octamer occurs only 69 times in the *C. trachomatis *D/UW3 genome, but never in succession.

### Phylogenetic analysis within *ompA *constant and variable domains

The nucleotide analysis above demonstrated that the variable and constant regions of the *ompA *gene are under different levels of selection (d_N_/d_S _= 0.34 and 0.04, respectively), which is in agreement with fact that each region is under different immunological and functional constraints. However, it is unknown if the disparity in synonymous nucleotide diversity between the variable and constant regions is due to recombination. The phylogenetic relationships among the nine regions were examined to determine if these regions evolved together or independently. If the phylogeny shows that they evolved together, then recombination was not a factor in generating the synonymous nucleotide diversity. Accordingly, trees were constructed from each of the nine variable and constant regions (Figure 1B-J). All of the trees were very similar to the *ompA *gene (Figure 1A), indicating the constant regions evolved in concert with the variable regions and independently of other regions in the genome. However, not all of the branching patterns in each region were identical; for example, strain F/IC-Cal3 did not group with strain G/UW-57 in the intermediate complex in constant domain 3 and variable domain 3 (Figure 1F, G). These differences coincide with the evidence of recombination with the *ompA *gene, and such heterogeneity would serve to explain some of the poor support (low bootstrap values) in an individual region. Because the phylogenies in all nine regions are highly similar, it implies that recombination did not shape the differences in synonymous nucleotide diversity between the variable and constant regions.

The synonymous and non-synonymous nucleotides in the *ompA *gene are under different selective constraints, and trees representing each class of sites from the *ompA *gene were constructed in order to compare their evolutionary histories (Additional file 2). These two trees were largely identical, indicating that the synonymous sites evolved in concert with the non-synonymous sites and are consistent with the *ompA *phylogeny.

### Divergence rates from MoPn

The murine *Chlamydia muridarum *MoPn, of which there is one known isolate, is a closely related common ancestor of the human *C. trachomatis *isolates. By comparing the divergence rates in orthologous regions between the human *C. trachomatis *isolates and the *C. muridarum *isolate, differences in mutation rates for each locus can be assessed (Table 3). The amount of non-synonymous replacements (K_A_) between mouse and human isolates is higher in the *ompA *gene than other regions, but this is expected as positive selection is known to occur in the variable domains. The rate of synonymous change (K_S_) in *ompA*, as well as the overall nucleotide substitution rate (D_XY_), is comparable between mouse and human isolates (Table 3).

**Table 3:** Divergence analysis of *C. trachomatis *human isolates and the murine *C. muridarum *MoPn isolate

Locus	K_A_	K_S_	K_A_/K_S_	D_XY_
676	0.056	1.461	0.04	0.12

680	0.064	0.694	0.09	0.16

*ompA*	0.112	0.981	0.11	0.24

CD1-5^A^	0.034	0.897	0.04	0.16

VD1-4^B^	0.569	1.396	0.41	0.72

682^C^	0.032	0.645	0.05	0.14

682^D^	0.019	1.170	0.02	0.16

687	0.094	1.070	0.09	0.24

HK^E^	0.052	0.915	0.06	0.19

^A^*ompA *constant domains 1-5

^B^*ompA *variable domains 1-4

^C^5' end of gene

^D^Middle of gene

^E^Housekeeping gene data from Brunelle and Sensabaugh, 2006 [8]

## Discussion

Examination of the *ompA *gene of *C. trachomatis *and its surrounding loci demonstrate an increase in nucleotide substitutions and differing phylogenetic histories compared to other regions of the genome. The increase in nucleotide substitution rates and the lack of any phylogenetic congruence in the regions flanking *ompA *are characteristic motifs of gene conversion. Gene conversion in bacteria is a non-reciprocal recombinatorial process in which one gene replaces another through strand invasion/displacement and repair via the recA-D proteins [27]. Such a process is common in bacteria; it occurs in at least 17 different genera [27], including *Chlamydia *[17,28,29]. Typically, gene conversion can serve one of two functions in the genome. First, it can promote diversification of antigenic outer membrane genes, as has been reported in species of *Anaplasma *[30], *Bartonella *[31], *Borrelia *[32,33], *Mycoplasma *[34], *Neisseria *[35,36], and *Treponema *[34]. Second, it can reduce nucleotide variation in functional genes by homogenizing homologs within a genome; there is evidence that all bacterial genomes with multiple-copy rDNA undergo gene conversion, which maintains the uniformity and the low-level of nucleotide diversity observed in the ribosomal gene sequences (concerted evolution) [37]. Both models, however, also lead to an increase in nucleotide diversity and indel events in the flanking regions [30,38,39]. This is what we observed in the *ompA *gene, supporting gene conversion of the *ompA *gene region.

Gene conversion alone does not explain the increase in polymorphisms within the *ompA *variable domains. An increase in the rate of amino acid replacements in the variable domains of the *ompA *gene compared to the constant domains is expected, as these regions are surface-exposed and are the main antigenic targets of the host immune system. However, the 3.8-fold increase in the rate of synonymous change in the variable domains relative to the constant domains is notable, especially considering that the constant domains contain more synonymous changes than most other genes in the genome. Though synonymous changes will hitchhike with the selection of favorable non-synonymous changes, this selection should occur for any polymorphism across the entire gene and not just in the variable domains. Nevertheless, in the *ompA *gene, the rate of synonymous change is clearly not evenly distributed. This disparity in the distribution of synonymous changes is not due to recombination of the variable domain because the phylogenetic trees for each constant and variable domain, in addition to the trees constructed from either synonymous or non-synonymous sites, are all congruent with each other and with the *ompA *gene. There appears to be some factor that increases the propensity of mutations in the variable regions (i.e. a mutational hotspot) that would explain the observations; a mutational hotspot has previously been demonstrated to occur in the *trpA *gene of *C. trachomatis *[40,41]. The high mutation rate observed in the synonymous sites in the variable domains compared to the constant domains appears to be evolutionarily conserved: the divergence analysis from the *C. muridarum *MoPn isolate indicates an equivalent level of synonymous change between the variable domains, the constant domains, and the neighboring genes.

It is not known what mechanism(s) promote the mutational increase in *ompA *and its neighboring loci relative to the rest of the genome. Codon bias causes a greater level of synonymous change in some genes as a way to increase transcriptional efficiency [42], but this would not occur in distinct segments of a single gene. Mutational bias associated with chromosomal location at the replication terminus [43] is also not a factor due to the fact that the *ompA *gene is only 60 kb from the origin of replication. One possible explanation is that a several-kilobase genomic region surrounding *ompA *has a unique secondary structure, which predisposes the region to increased mutations and decreased access to DNA repair machinery. While the whole region would be subject to increased mutations, more mutations could become fixed in the *ompA *gene if it had fewer functional constraints than the neighboring loci. In addition, the level of *ompA *expression may play a role: because *ompA *is one of the most highly expressed genes in *C. trachomatis *[44], it spends more time than other genes being single stranded and therefore may have a higher propensity of acquiring mutations in this state [45,46]. Mutations are more likely to occur in the non-template strand because it is single-stranded and not protected by the replication bubble, and is therefore more prone to DNA damage and deamination that can lead to nucleotide replacements, insertions, or deletions [47-50]. Coupled with the potential translesion activity of DNA polymerase I [51] and positive selection, these mutations in *ompA *could become permanent.

## Conclusions

The discordant phylogenetic associations of the loci neighboring the *ompA *gene, along with the increased rate of nucleotide diversity in these regions, can be ascribed to recombination of the *ompA *gene via gene conversion. In addition, the high synonymous substitution rate within the variable domains of *ompA *appears to be the result of an unidentified influence that generates an increase in mutations within the gene or genomic region. Together, the increased mutation rate in the *ompA *gene acts in concert with positive selection and recombination to promote the high degree of variability observed in MOMP in order to evade immune detection.

## Methods

### Source of isolates

Nineteen serovars were originally obtained through Dr. J. Schachter, University of California, San Francisco (A/Har-1, A/Har-13, B/Tunis 864, B/Har-36, Ba/Apache 2, C/TW-3, C/Har-32, D/IC-Cal 8, E/Bour, F/IC-Cal 3, G/392, G/UW-57, H/580, Ia/870, J/UW-36, K/UW-31, L1/440, L2/434, L3/404). Three additional strains were provided by Dr. R. S. Stephens, University of California, Berkeley (B/TW-5, D/UW-3, I/UW-12).

### DNA isolation from culture

DNA was isolated using the standard proteinase K digestion, phenol-chloroform-isoamyl extraction, and ethanol precipitation [52].

### Genes of interest

The *ompA *gene sequences from these isolates are available in GenBank [GenBank: DQ064279-DQ064299] [8]. Primers were designed to amplify seven regions within 10 kb of either end of the *ompA *gene, and each PCR product was purified using ExoSAP-IT (GE Healthcare, Piscataway, NJ), verified on a 2% agarose gel, and sequenced on an ABI 377 sequencer using Big Dye 3 terminator chemistry (Applied Biosystems, Foster City, CA). Sequences were deposited in GenBank [GenBank: DQ063870-DQ063890; DQ063912-DQ064017; DQ064039- DQ064059]. Orthologous regions from the Mouse Pneumonitis (MoPn) biovar of *Chlamydia muridarum *strain Nigg were retrieved from the genome sequence [GenBank: AE002160] [53].

### Alignment and analysis

The nucleotide sequences for each locus were aligned using ClustalX [54] employed in BioEdit http://www.mbio.ncsu.edu/BioEdit/bioedit.html. For coding regions, nucleotide sequences were translated to their amino acid sequences, aligned, and then translated back to their nucleotide sequences within the alignment. The software package MEGA4 [55] was used to calculate the following from the human isolates of *C. trachomatis*: the number of variable nucleotide sites in each locus; the number of non-synonymous substitutions per non-synonymous site (d_N_) and the number of synonymous substitutions per synonymous site (d_S_) using the Nei-Gojobori method and Jukes and Cantor correction with pairwise deletions for alignment gaps; and the ratio of transitions to transversions using pairwise deletion of alignment gaps (ts/tv). MEGA4 was also used to construct phylogenetic trees using the Maximum Composite Likelihood neighbor-joining method and pairwise deletions for alignment gaps. The only exception was the two trees built from either the synonymous or non-synonymous sites of *ompA*; for these analyses, the Nei-Gojobori neighbor-joining method was employed with the Jukes and Cantor correction and pairwise deletions for alignment gaps. All trees were supported by 1000 bootstrap replicates. The software package DnaSP v5 [56] was used to calculate the following divergence values with Jukes and Cantor correction between the human isolates of *C. trachomatis *and the MoPn isolate: non-synonymous substitutions per non-synonymous site (K_A_), synonymous substitutions per synonymous site (K_S_), and average number of substitutions per nucleotide site between populations (D_XY_).

## Competing interests

The authors declare that they have no competing interests.

## Authors' contributions

BWB and GFS designed the study, BWB performed experiments, and BWB and GFS analyzed data and wrote the manuscript. Both authors read and approved the final manuscript.

## Supplementary Material

Additional file 1 **Figure S1**. Phylogenetic analyses of the *C. trachomatis ompA *gene flanking regions. Click here for file

Additional file 2 **Figure S2**. Phylogenetic analyses of the synonymous and non-synonymous sites of the *ompA *gene. Click here for file
