# Supplementary material for: Nucleotide and phylogenetic analyses of the Chlamydia trachomatis ompA gene indicates it is a hotspot for mutation
Source: BMC Res Notes. 2012 Jan 20;5:53. doi: 10.1186/1756-0500-5-53 (PMC3296649; doi:10.1186/1756-0500-5-53)

Additional Figure 1. Phylogenetic analyses of the *C. trachomatis ompA* gene flanking regions. Phylogenetic trees of the *C. trachomatis ompA* gene flanking regions (A-G) were constructed using the Maximum Composite Likelihood neighbor-joining method with pairwise deletions of alignment gaps. Results of 1000 bootstrap replicates are reported for each node.

A. CT676

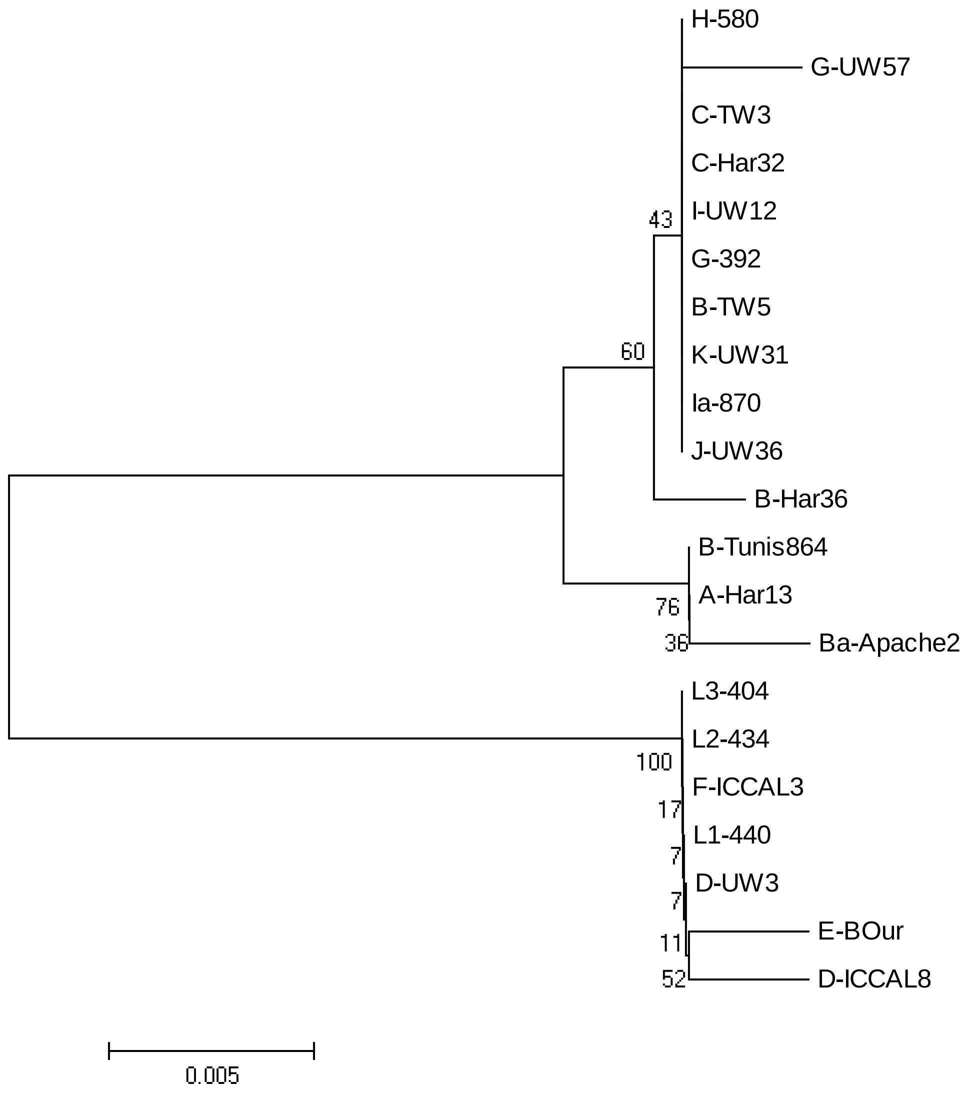

B. CT680 (*rs2*)

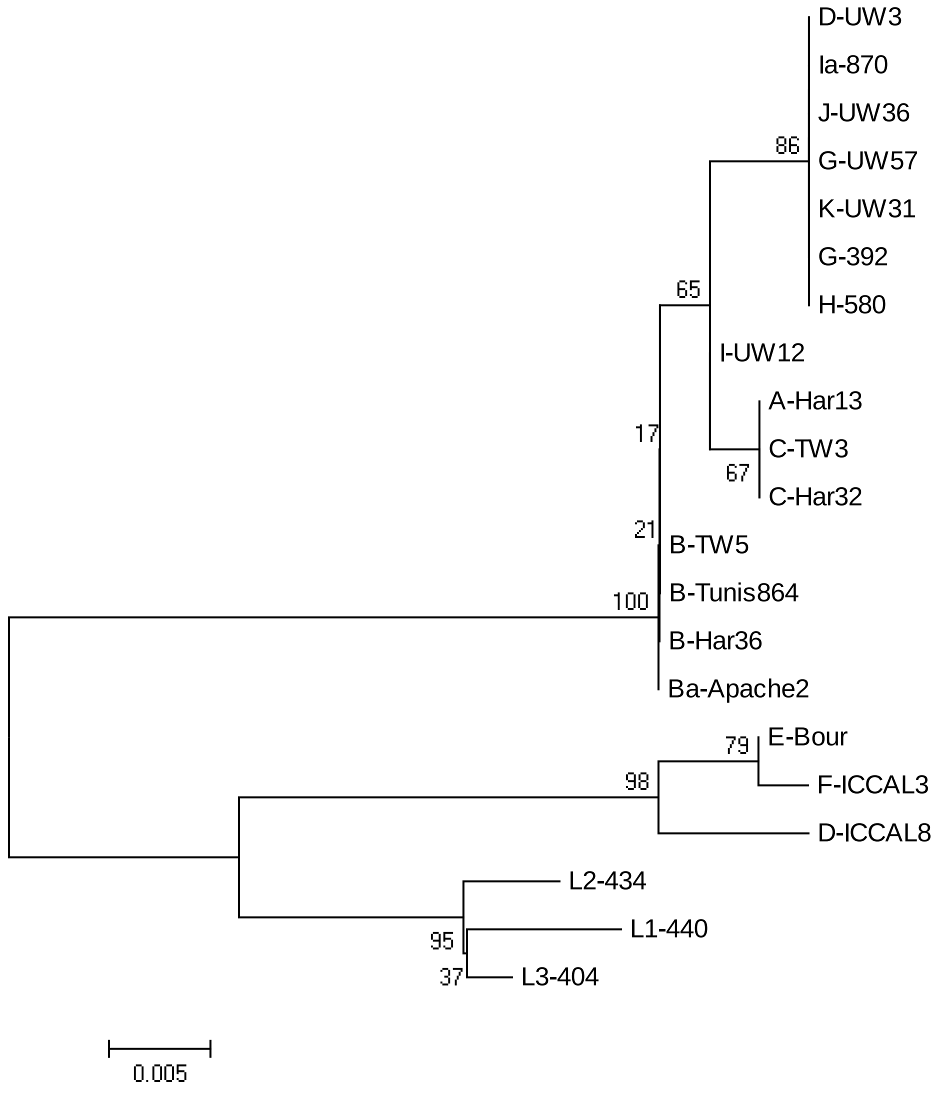
D-UW3
Ia-870
J-UW36
G-UW57
K-UW31
G-392
H-580
I-UW12
A-Har13
C-TW3
C-Har32
B-TW5
B-Tunis864
B-Har36
Ba-Apache2
E-Bour
F-ICCAL3
D-ICCAL8
L2-434
L1-440
L3-404
86
65
17
67
21
100
79
98
95
37
0.005

C. CT680-1

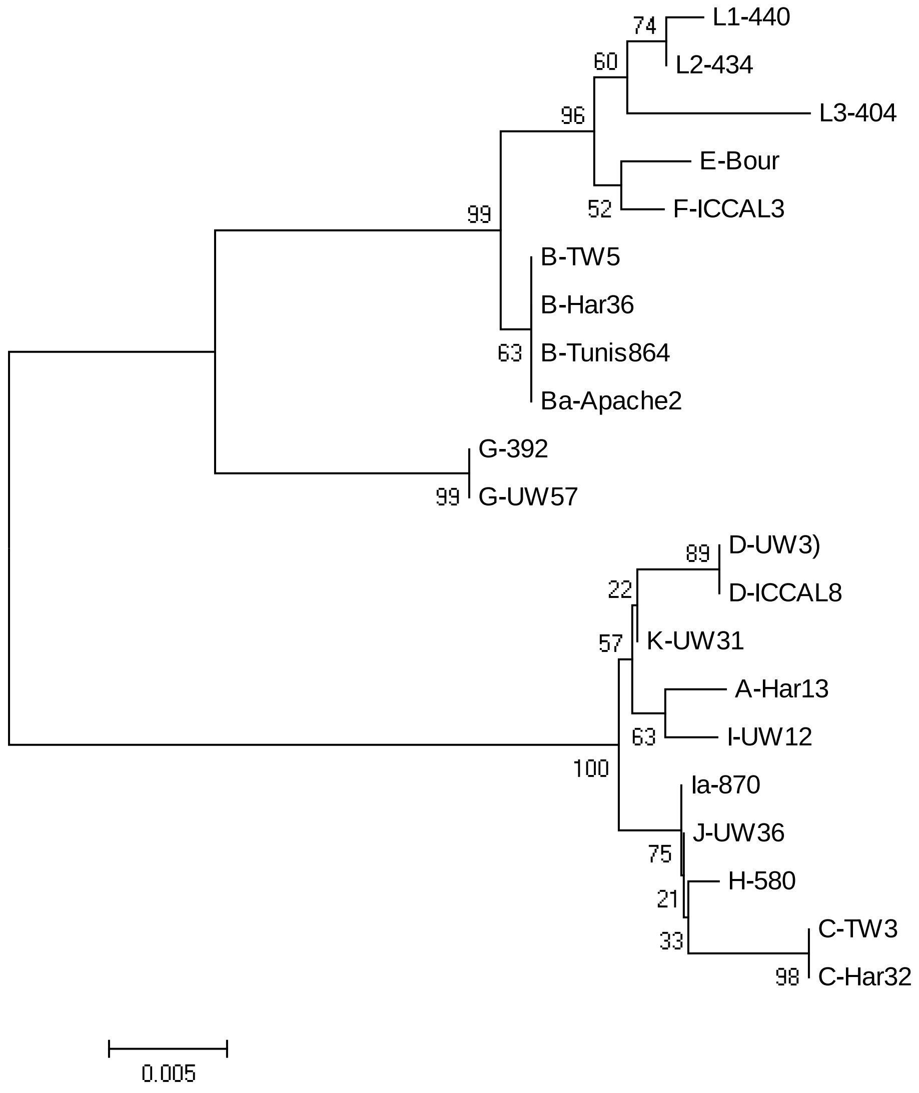

L1-440
L2-434
L3-404
E-Bour
F-ICCAL3
B-TW5
B-Har36
B-Tunis864
Ba-Apache2
G-392
G-UW57
D-UW3)
D-ICCAL8
K-UW31
A-Har13
I-UW12
Ia-870
J-UW36
H-580
C-TW3
C-Har32
74
60
96
99
52
63
99
89
22
57
63
100
75
21
33
98
0.005

D. CT681-2

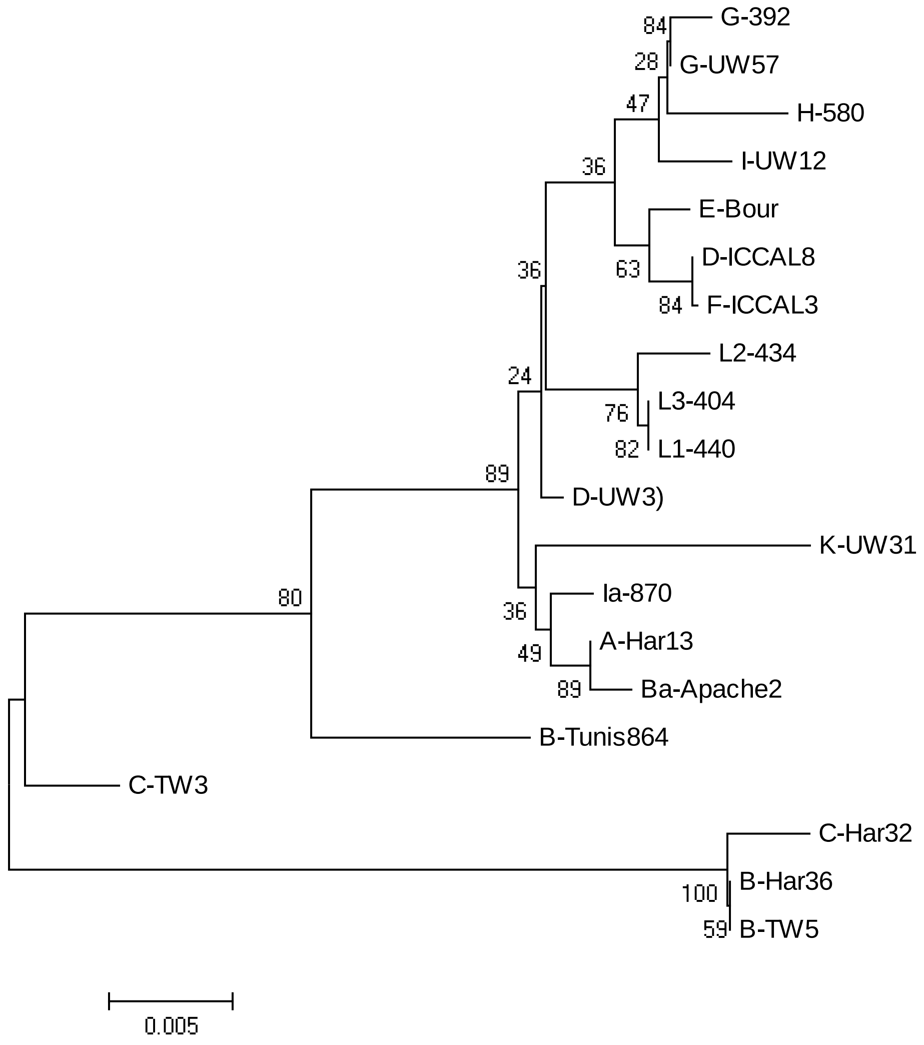
G-392
G-UW57
H-580
I-UW12
E-Bour
D-ICCAL8
F-ICCAL3
L2-434
L3-404
L1-440
D-UW3)
K-UW31
Ia-870
A-Har13
Ba-Apache2
B-Tunis864
C-TW3
C-Har32
B-Har36
B-TW5
84
28
47
36
63
84
36
24
76
82
89
36
49
89
80
100
59
0.005

E. CT682 (5' end; *pbpB*)

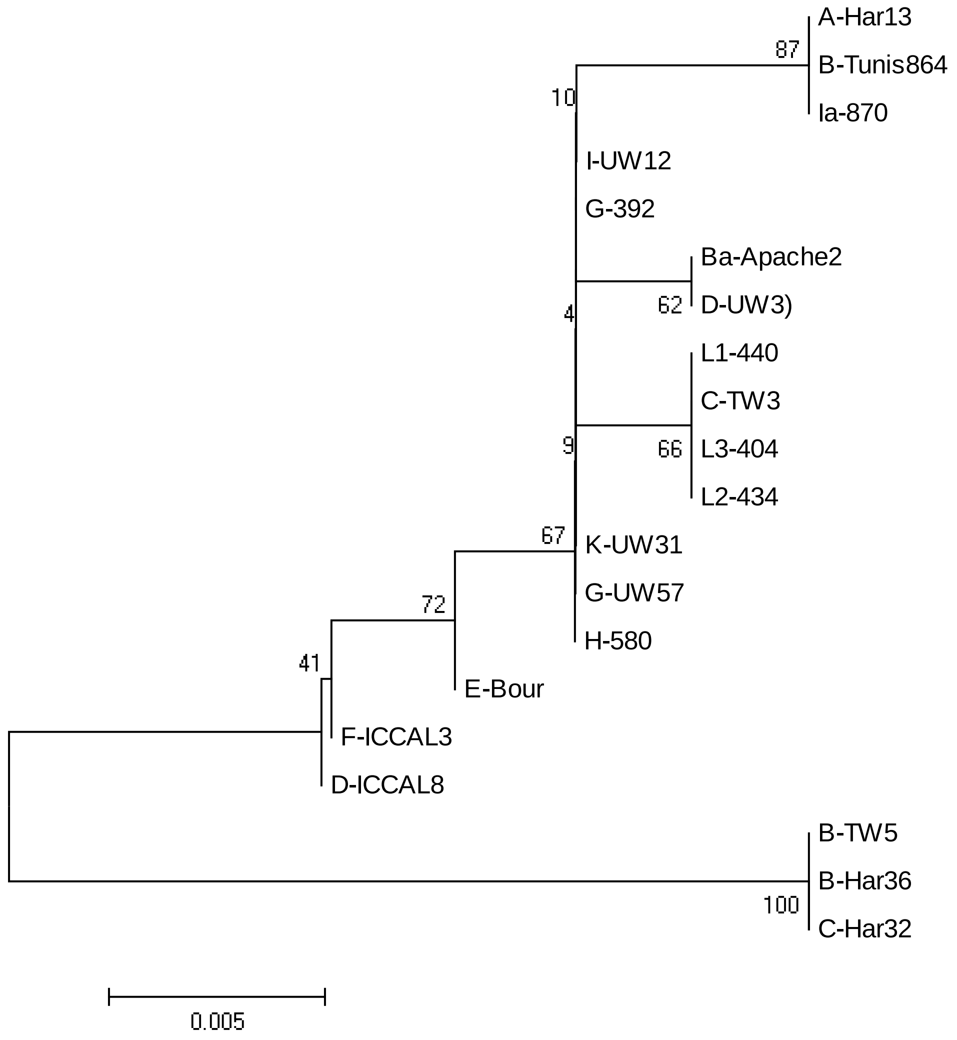

F. CT682 (middle; *pbpB*)

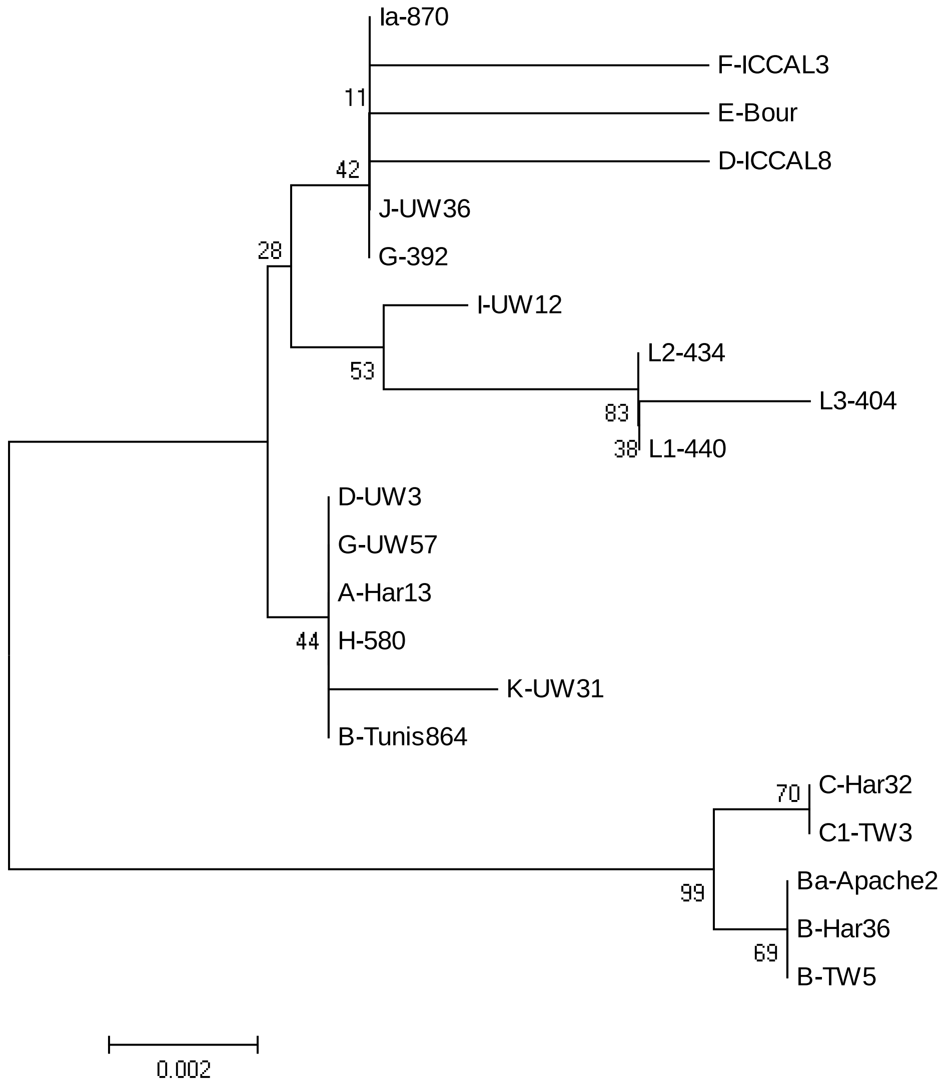

G. CT687 (*yfhO2*)

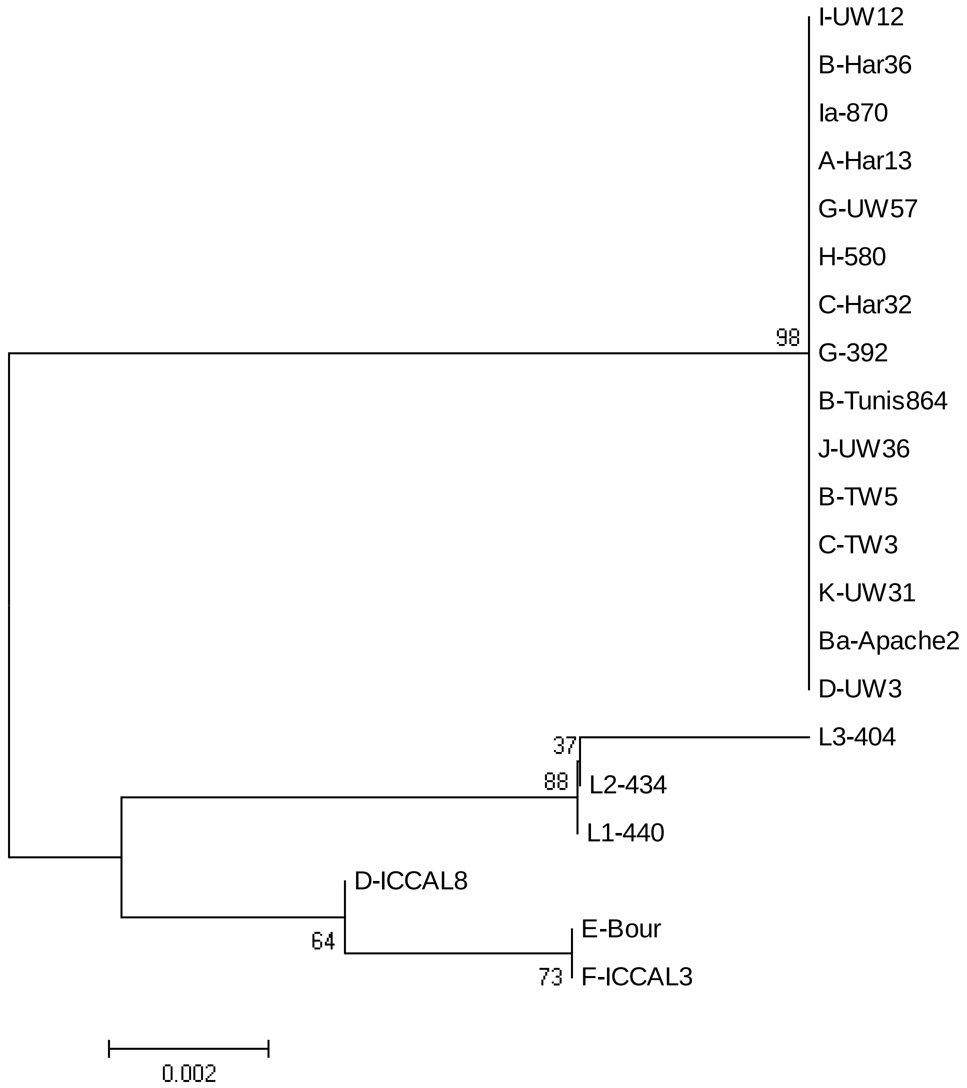

Supplement: Additional file 1 — Figure S1. Phylogenetic analyses of the C. trachomatis ompA gene flanking regions. [file 1756-0500-5-53-S1.PDF]
