# Supplementary material for: Nucleotide and phylogenetic analyses of the Chlamydia trachomatis ompA gene indicates it is a hotspot for mutation
Source: BMC Res Notes. 2012 Jan 20;5:53. doi: 10.1186/1756-0500-5-53 (PMC3296649; doi:10.1186/1756-0500-5-53)

Additional Figure 2. Phylogenetic analyses of the synonymous and non-synonymous sites of the *ompA* gene. Phylogenetic trees of both the synonymous and non-synonymous sites of the *ompA* gene were constructed using the Nei-Gojobori neighbor-joining method with Jukes and Cantor correction and pairwise deletions of alignment gaps. Results of 1000 bootstrap replicates are reported for each node.

A. synonymous sites

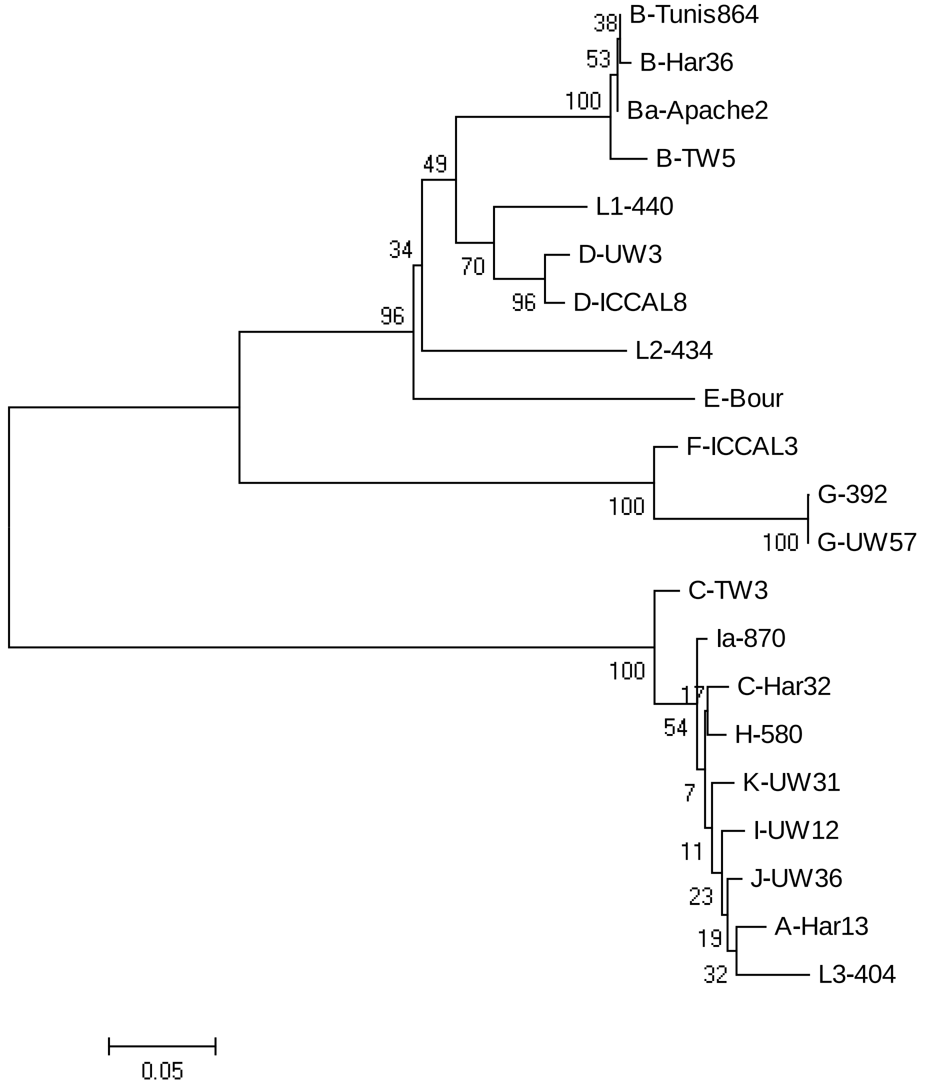

B. non-synonymous sites

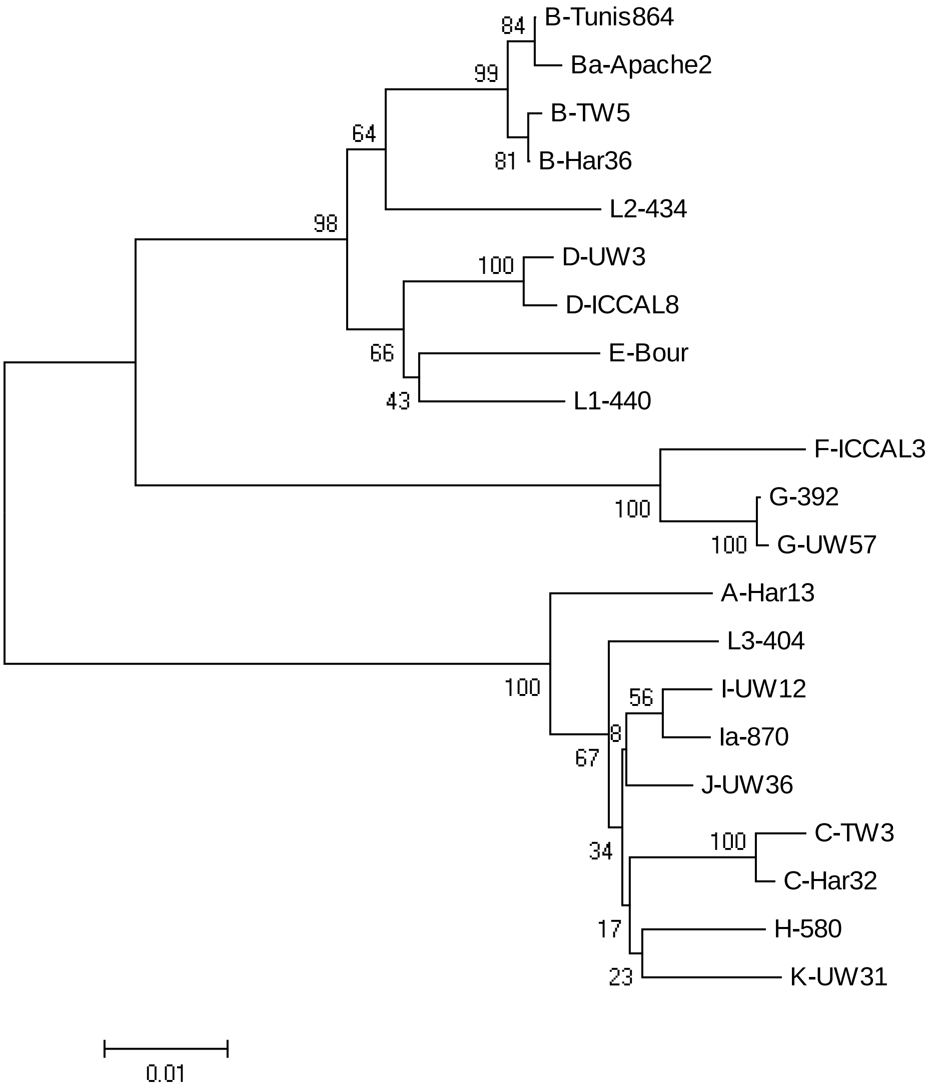

Supplement: Additional file 2 — Figure S2. Phylogenetic analyses of the synonymous and non-synonymous sites of the ompA gene. [file 1756-0500-5-53-S2.PDF]
